# Prevalence of enteroviruses in healthy populations and excretion of pathogens in patients with hand, foot, and mouth disease in a highly endemic area of southwest China

**DOI:** 10.1371/journal.pone.0181234

**Published:** 2017-07-13

**Authors:** Qiang Wu, Xiaoqing Fu, Lili Jiang, Rusong Yang, Jianping Cun, Xiaofang Zhou, Yongming Zhou, Yibing Xiang, Wenpeng Gu, Jianhua Fan, Hong Li, Wen Xu

**Affiliations:** 1 Yuxi City Center for Disease Control and Prevention, Hongta District, Yuxi City, Yunnan, People’s Republic of China; 2 Yunnan Provincial Centers for Disease Control and Prevention, Kunming, Yunnan, People’s Republic of China; 3 Xishuang Banna Autonomous Prefecture Centers for Disease Control and Prevention, Jinghong City, Yunnan, People’s Republic of China; University of Florida, UNITED STATES

## Abstract

Etiological carriers and the excretion of the pathogens causing hand, foot, and mouth disease (HFMD) in healthy persons, patients, and asymptomatic persons infected with HFMD as ongoing infection sources may play an important role in perpetuating and spreading epidemics of HFMD. The aims of this study were to determine the carrier status of EV-A71 and CV-A16 in healthy populations, as well as the duration of EV-A71 and CV-A16 shedding in the stools of HFMD patients in an epidemic area of southwest China. A cross-sectional study and a follow-up study were conducted in three HFMD endemic counties of Yunnan Province. Six hundred sixty-seven healthy subjects were recruited to participate in the cross-sectional study, and two stool specimens were collected from each subject. Among the healthy subjects, 90 (13.5%) tested positive for viral isolation, but neither EV-A71 nor CV-A16 was detected in healthy individuals. Of the 150 patients with probable HFMD, 55.3% (83/150) tested positive for viral isolation with presented serotypes such as EV-A71 (51.81%, 43/83), CV-A16 (32.53%, 27/83), other EVs (13.25%, 11/83), and mixed EV-A71 and CV-A16 (2.41%, 2/83). The longest duration of EV-A71 and CV-A16 shedding in stool specimens from patients with HFMD was >46 days after onset. The positive rate of EV-A71 in the stool specimens of confirmed patients dropped to 50% by the end of the third week, and the same occurred with CV-A16 by the end of approximately the seventh week after onset. Although carriers of major causative agents of HFMD in healthy populations are fewer in number, the prolonged shedding of pathogens in patients with HFMD may serve as an important factor in perpetuating and spreading HFMD epidemics.

## Introduction

Hand, foot, and mouth disease (HFMD) is a common acute enteric infectious disease among young children, particularly those <5 years of age [1]. The causal agents of HFMD involve a number of human enteroviruses (HEVs), including HEV A and B species, though EV-A71 (Enterovirus 71) and CV-A16 (Coxsackievirus A16) are the two major pathogens that cause HFMD [2–4]. The disease is self-limiting, with mild symptoms for most patients, but severe complications including death can arise for some patients, particularly those who are infected with EV-A71 [3, 5, 6]. Outbreaks or epidemics of HFMD have occurred in many countries around the world, and the Asia-Pacific region has been severely affected by HFMD in recent years [3, 7–9].

Before 2007, sporadic cases and rare small outbreaks of HFMD were reported in mainland China [10, 11]. Large-scale outbreaks of HFMD occurred in 2007 and 2008 in the Shandong and Anhui provinces of China, respectively [12, 13]. Since then, HFMD has been widespread and endemic in mainland China, with high and annually increasing incidence [1, 14], which is associated with more severe cases and death. Extensive and continual HFMD epidemics in some areas of mainland China constitute a major public health concern.

The source of infection, route of transmission, and herd susceptibility are three basic necessary factors for communicable diseases to become epidemics, and these factors also apply to HFMD. Although epidemics of HFMD are affected by various factors, humans are the only known natural hosts of HEVs [3], and the prolonged excretion of pathogens in patients with HFMD is a very important source of infection for HFMD epidemics [15]. The carriers of HFMD pathogens among healthy persons have also been highlighted as major sources of infection in HFMD epidemics in a variety of previous studies, each with small sample sizes, different specimen types and different age groups [16–19]. The aims of this study were to determine the prevalence of EVs in healthy populations and the excretion of pathogens in patients with HFMD in areas with the same high rates and the same period of HFMD epidemics to gain an overview of the infectious source of HFMD and to evaluate the infectious source, control and prevention of HFMD.

## Materials and methods

### Study design and setting

We performed a cross-sectional study and a follow-up study, and relevant information about subjects was collected with face-to-face interviews using structured questionnaires. Additionally, stool specimens were collected from healthy persons and patients with probable HFMD.

This study was conducted in the city of Yuxi in Yunnan Province from May to July 2013. Yuxi is divided into nine counties and has a total population of approximately 2,300,000 residents. Within the city, three counties with high HFMD incidence (Tonghai, Xinping and Yuanjiang) were included in the study constituting a total population of 758,000 people. The HFMD incidence rates in Tonghai, Xinping, and Yuanjiang counties were 382.34/100,000, 246.57/100,000, and 478.74/100,000 in 2013, respectively.

### Definition

#### HFMD probable case

According to diagnostic criteria defined previously by the Ministry of Health, mild cases of HFMD were defined as patients with vesicular lesions on their palms, feet, and mouth and with or without fever, whereas severe cases were defined as HFMD accompanied by neurological or cardiopulmonary complications. Herpangina was not included in this definition.

(http://www.chinacdc.cn/jkzt/crb/bl/szkb/jszl_2275/200906/t20090612_24707.html).

#### Healthy person

A healthy person was defined as a person without any symptoms or history of HFMD during the study period.

#### Two stool specimens

Two stool specimens were collected from each healthy person and each patient with a probable HFMD case, with an interval of 24–48 h between collections.

### Study subject recruitment and sample size

To be eligible for the study, the study subjects were required to be native residents. Healthy persons without any symptoms or history of HFMD during the study period were recruited to participate in the study. Healthy persons having any symptoms of HFMD during the study period were excluded. Patients with a probable HFMD case who were making their second or subsequent visit for the same episode of HFMD or who were diagnosed with another disease during the study period were excluded.

In the cross-sectional study, the prevalence of EVs was approximately 10% in the healthy population based on previous data [20]. We assumed that 10% of our subjects would be carriers of EVs in the study. In addition, previous data shows that EVs are detected in the stool specimens of approximately 80% of patients with HFMD after illness [16]. In maintaining a significance level of 0.05 and a power of 90% and considering no responses and loss to follow-up, the total sample sizes in the cross-sectional study and the follow-up study were calculated to be no less than 650 healthy persons and 150 patients with probable HFMD cases, respectively, in the three counties.

### Collection of data and stool specimens

To sample healthy persons, three townships in each county of the study were selected using simple random sampling. After all of the administrative villages in the selected townships were numbered, one administrative village was then selected using simple random sampling. A list of residents’ names was obtained from the selected administrative villages, and these residents were assigned numbers. Approximately, one-third of the total 215 subjects were sampled in each selected administrative village using simple random sampling with SPSS software (version 19.0, Armonk, NY). If not enough samples were obtained from one selected administrative village, more samples were obtained from a neighboring village using the same method. After the list of healthy persons’ names was determined, research assistants visited those specific households in the village to collect data (including essential information such as gender, date of birth, family address, and history of illness) using questionnaires. They also distributed stool collection kits to each house in the evening. Each selected healthy person provided two stool specimens. If selected healthy persons were not at home or ineligible for the study, they were excluded, and the recruiter enrolled the sample in this study.

In the study area, the county hospital was appointed as the designated hospital to receive patients with HFMD by the local health authority. Probable cases of HFMD were identified according to the national guidelines for the control and prevention of HFMD issued by the Ministry of Health.

(http://www.chinacdc.cn/jkzt/crb/bl/szkb/jszl_2275/200906/t20090612_24707.html).

The first sample of stool was collected immediately within 3 days after onset when a patient was diagnosed as a probable HFMD case, and the second sample of stool was taken ≤ 48 h later. Subsequently, six stool samples were collected once a week for 6 weeks. All specimens were transported to the laboratory at 4–8°C and stored at -20°C until tested.

### Laboratory testing

#### Virus isolation

The isolation of the virus was performed following the national guidelines for the control and prevention of HFMD issued by the Ministry of Health. (http://www.chinacdc.cn/jkzt/crb/bl/szkb/jszl_2275/200906/W020130106522855465929.pdf). Human rhabdomyosarcoma cells (RD), human larynx carcinoma cells (Hep-2), and L20 B cells were used to isolate the virus and were provided by the National Polio Laboratory.

#### Molecular typing

Positive cell cultures were repeatedly frozen and thawed three times. Then, RNA was extracted from each positive culture and 10% of stool suspension for relevant negative-culture samples (healthy persons, probable HFMD cases and confirmed cases) using RNeasy Mini Kits (QIAGEN, Hilden, Germany) following the manufacturer’s instructions. To identify the EV type in positive cultures, a reverse transcription-PCR (RT-PCR) based on amplification of a partial sequence of EVs was performed using primers for EVs (A, B, C, and D groups), EV-A71, CV-A16, and polio (Table 1) [12, 21–23]. The positive and negative controls were set up. Non-infectious control RNA was positive control. Distillation-Distillation H_2_O was negative control. The RT-PCR products were visualized on 1.5% agarose gels containing 0.1 μL/mL Gelview under a UV transilluminator. The amplicons were subjected to sequencing and BLAST analysis. The detection of EV, EV-A71, and CV-A16 for a HFMD diagnosis was performed with multiplex real-time PCR using a kit for the detection of CV-A16, EV-A71, and universal EV nucleic acid (Fluorescent PCR method, Jiangsu, China). The types of EVs isolated from probable cases of HFMD were identified by molecular typing.

**Table 1:** Specific primer amplification sequences of the enterovirus VP1 region.

Specificity	Primer	Sequence (5’-3’)
EV-A71	EV-A71-VP1-S	GCAGCCCAAAAGAACTTCAC
	EV-A71-VP1-A	AAGTCGCGAGAGCTGTCTTC
CV-A16	CV-A16-VP1-S	ATTGGTGCTCCCACTACAGC
	CV-A16-VP1-A	GCTGTCCTCCCACACAAGAT
Enterovirus universal primer	292	MIGCIGYIGARACNGG
	222	CICCIGGIGGIAYRWACAT
Enterovirus A	487	ATGTWYGYICCICCIGGIGCNCC
	488	GTIGGRTAICCITCITARAACCAYTG
Enterovirus B	491	ATGTAYRTICCICCIGGNGG
	492	GGRTTIGTIGWYTGCCA
Enterovirus C	495	ATGTAYRTICCICCIGGIGCNCC
	496	CCRTCITARAARTGISIRTANGC

### Data analysis

**Fig 1:**
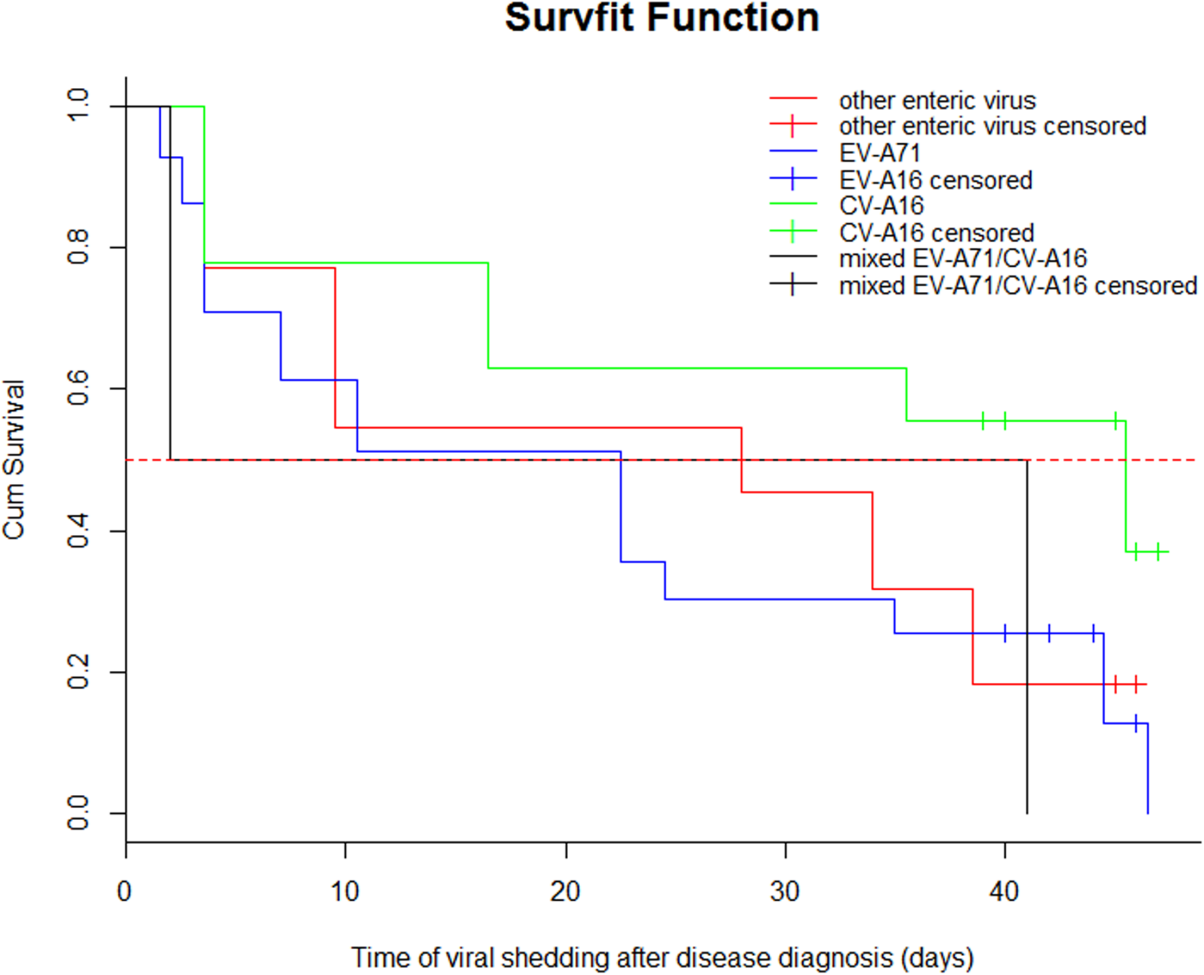
The survival curves of the excretion of different enterovirus types among confirmed HFMD patients. The positive rate of virus isolation of EV-A71, CV-A16, other EVs, and mixed EV-A71/CV-A16 in serially collected stool samples from confirmed HFMD patients. The red dashed line represents 50% survival probability indicator. Other virus: time, 38.5 days; n. event, 1.5; survival, 0.182; 95% CI: 0.0519, 0.637. EV-A71: time, 44.5 days; n. event, 3.00e+00; survival, 0.128; 95% CI: 0.0495, 0.330. CV-A16: time, 45.5 days; n. event, 2; survival, 0.370; 95% CI: 0.192, 0.716. Mixed EV-A71/CV-A16: time, 2days; n. event, 1; survival, 0.5; 95% CI: 0.125, 1.

Data analysis was performed with R software (version 3.3.2) and SPSS (version 19.0, Armonk, NY). Chi-squared and Fisher’s exact tests were used for the categorical data analysis. The endpoint of virus shedding is the last positive-sample date. However, interval-censored types have been considered, the middle endpoint between the last positive culture date and the first negative culture date was applied to count cumulative survival, and the survfit function of the survival package was employed to compare the differences in duration of virus shedding among the four different pathogen groups (Fig 1). All healthy persons were divided into five age groups according to the age distribution of patients with HFMD [24]. Additionally, P<0.05 indicated a significant difference.

### Ethnical approval

The study was approved by the Ethical Review Committee of the Yunnan Provincial Centers for Disease Control and Prevention. Informed verbal or written consent was obtained from the subjects or their parents/guardians before collecting samples. The human sample collection and detection protocols were performed in accordance with relevant guidelines and regulations. IRB (The Institutional Review Board) approved the use of oral consent, and the information of consent contained the aim of the study, the usage of the patient’s samples, personal confidentiality agreement *etc*. The informed consents were oral for all the participants, because the samples were too large; we couldn’t get all the written ones. We recorded the participant consent by signature on the list of the personal confidentiality agreement. And we revised this part of manuscript in the ethics statement.

## Results

### Prevalence and types of EVs in the healthy population

During the study period, 667 healthy persons from 2 months to 63 years old [average age: 7.67±23.45 years, (M±Q)] were recruited with a combined collection of 667 pairs of stool specimens (1334 stool specimens). Of these participants, 278 (41.68%) were male, and 389 (58.32%) were female. All of them were divided into five age groups (<1, 1-<3, 3-<5, 5-<10, and ≥10 years old) (Table 2). Among the 667 healthy persons, 90 (13.5%, 90/667) tested positive for viral isolation in the stool specimens, including 83 persons who tested positive for a single type of EV, three who tested positive for two types of EV, one person who tested positive for a single type of EV and mixed adenovirus, and three who tested positive for adenovirus. The positive rates of viral isolation in males and females were 15.8% (44/278) and 11.8% (46/389), respectively. No significant differences were identified in the positive rates of viral isolation in terms of gender distribution (P>0.05). The positive rates of viral isolation for the age groups of <1 year, 1 to 3 years, 3 to 5 years, 5 to 10 years, and ≥10 years were 23.5% (12/51), 18.3% (19/104), 18.3% (21/115), 15.5% (13/84) and 8.0% (25/313), respectively. The positive rates of viral isolation among each of the different age groups were significant (P<0.05); younger age groups had higher positive rates of EV isolation (P<0.05) (Table 2). For viral isolation of the two stool specimens, the numbers of persons with positive viral isolation in both specimens, the first specimen only, and the second specimen only were 40, 29, and 21, respectively.

**Table 2:** Number of positive viral isolates from healthy persons by gender and age group.

Age group	Male		Female		Total		
(year)	N (%)	No. of healthy persons with positive viral isolation (%)	N (%)	No. of healthy persons with positive viral isolation (%)	N (%)	No. of subjects with positive viral isolation (%)	Number of virus strains
<1	20 (7.2)	8 (40.0)	31 (8.0)	4 (13.8)	51 (7.6)	12 (23.5)	23**
1 to <3	61 (21.9)	12 (19.7)	43 (11.1)	7 (15.9)	104 (15.6)	19 (18.3)	32*
3 to <5	62 (22.3)	14 (22.6)	53 (13.6)	7 (13.2)	115 (17.2)	21 (18.3)	33*
5 to <10	37 (13.3)	6 (16.2)	47 (12.1)	7 (14.6)	84 (12.6)	13 (15.5)	19
≥10	98 (35.3)	4 (4.1)	215 (55.3)	21 (9.8)	313 (46.9)	25 (8.0)	30
total	278 (100.0)	44 (15.8)	389 (100.0)	46 (11.8)	667 (100.0)	90 (13.5)	137

**Two persons with mixed strain

*One person with mixed strain.

In total, 131 EV strains and six adenovirus strains were isolated from two stool specimens each from 90 healthy persons, with a rate of EV isolation of 9.8% (131/1334). Additionally, EV species B had 104 strains (79.4%, 104/131), with the most dominant serotypes being E(Echovirus)-25 (18.3%, 24/131) and CV-B (Coxsackievirus B)2 (11.5%, 15/131), followed by other serotypes including E-1, E-6, E-11-E-14, E-19, E-30, CV-B2, CV-B4, and CV-B5. Similarly, EV species C had 24 strains (18.3%, 24/131), of which the majority were PV(Poliovirus)/Sabin (14.5%, 19/131), and other serotypes included CV-A(Coxsackievirus A)13 and CV-A24. Moreover, EV species A (2.3%, 3/131) had few detected strains, including CV-A2 and CV-A4. In addition, other EVs (55.7%, 73/131) related to HFMD were CV-A2, CV-A4, E-13, E-19, E-25, E-30, CV-B2, CV-B4, and CV-B5 (Table 3).

**Table 3:** Distribution of enterovirus types in stool specimens of healthy persons.

Species (%)	Serotype	No. of virus (%)	Pathogen of HFMD (%)
Enterovirus A	CV-A2	1 (0.8)	1 (0.8)
(3.19)	CV-A4	2 (1.6)	2 (1.6)
Enterovirus B	E-1	1 (0.8)	-
(74.47)	E-6	12 (9.1)	-
	E-11	2 (1.6)	-
	E-12	6 (4.6)	-
	E-13	10 (7.6)	10 (7.6)
	E-14	13 (9.9)	-
	E-19	4 (3.0)	4 (3.0)
	E-25	24 (18.3)	24 (18.3)
	E-30	7 (5.3)	7 (5.3)
	CV-B2	15 (11.4)	15 (11.4)
	CV-B4	4 (3.0)	4 (3.0)
	CV-B5	6 (4.6)	6 (4.6)
Enterovirus C	PV-1 (Sabin)	14 (10.7)	-
(18.09)	PV-2 (Sabin)	3 (2.3)	-
	PV-3 (Sabin)	2 (1.6)	-
	CV-A13	1 (0.8)	-
	CV-A24	4 (3.0)	-
Total	-	131* (100.0)	73 (55.7)

*Six adenovirus rejected.

CV-A = coxsackievirus A; CV-B = coxsackievirus B; E = echovirus; and PV = poliovirus.

RT-PCR was performed on 1154 negative-culture samples (stool suspensions) of healthy persons. EV RNA was detected in 4 stool specimens (4/1154) and identified as CV-A1 (2), E-16 (1) and PV-1 (Sabin) (1).

### Excretion of pathogens in patients with HFMD

In total, of the 150 probable HFMD cases, 85 (56.7%) of them were male and 65 (43.3%) were female, with an average age of 2.85±1.24 years (*—x*±s) (range, 0.8–7 years). Collectively, 1,200 stool specimens were collected from 150 probable cases of HFMD. Among the probable HFMD cases, 83 cases with age ranging from 6 months to 7 years old (average age 2.94 ± 1.29 years, *—x*±s), of whom 48 (57.8%) were male and 35 (42.2%) were female, were positive for EV isolation (55.3%, 83/150) in stool samples within 3 days after onset. They were divided into five age groups: 0 to 1 year (19, 22.9%), 2 years (14, 16.9%), 3 years (25, 30.1%), and 4 years (25, 30.1%). Serotypes presented in EVs isolated from 83 HFMD cases were EV71 (51.81%, 43/83), CV-A16 (32.53%, 27/83), other EVs (13.25%, 11/83), and mixed EV71 and CV-A16 (2, 2.41%). Eleven other EVs (non-EV71/CV-A16) associated with HFMD in this study included CV-A10 (4), CV-A6 (3), CV-A4 (1), CV-A12 (1), CV-A9 (1) and E25 (1).

Furthermore, 74.4% (32/43) of EV-A71 cases, 77.8% (21/27) of CV-A16 cases, 81.8% (9/11) of other cases (non-EV-A71/CV-A16), and 100% (2/2) of mixed EV-A71/CV-A16 cases displayed intermittent virus excretion in the stool samples.

Among the 83 patients with HFMD who presented virus excretion in their stools after onset, the observation periods of virus shedding for 43 patients with EV-A71 infection, 27 patients with CV-A16 infection, 11 patients with other EVs infection, and 2 patients with mixed EV-A71/CV-A16 infection was 46 days, 47 days, 46 days, and 41 days, respectively. A total of 22 patients were censored, including 7 patients infected with EV-A71, 13 patients infected with CV-A16, and 2 patients infected with other EVs. The shortest duration of EV-A71, CV-A16, other EVs, and mixed EV-A71/CV-A16 shedding was at ≥1 days after onset. The longest duration of EV71-positive, CV-A16-positive, other EV-positive and EV-A71/CV-A16-positive shedding in feces was > 46 days and 41 days for mixed EV-A71/CV-A16 cases after onset. Mean survival times of viral shedding were 20.40 days (95% CI: 15.44, 25.35), 30.30 days (95% CI: 23.46, 37.13), 24 days (95% CI: 12.41, 35.59) and 21.50 days (95% CI: 0.00, 59.72) for EV-A71, CV-A16, other EVs, and mixed EV-A71/ CV-A16. Median survival times of viral shedding were 20 days (95% CI: 4.58, 35.24), 40 days (95% CI: -, -; there were a lot of censored in data for CV-A16 group, 95% CI could not be obtained.), 27 days (95% CI: 0.026, 53.97), and 21.50 day (95% CI: -, -; there were only two cases, 95% CI could not be obtained.) for persons infected with EV-A71, CV-A16, other EVs and EV-A71/ CV-A16 mixed, respectively. There were differences in the median survival time between EV-A71, CV-A16, other EVs, and EV-A71/CV-A16 mixed shedding (Log-rank test, χ^2^ = 8.577, P = 0.035). Approximately 51.9% of patients with CV-A16, 25.6% of patients with EV-A71, and 27.3% of patients with other EVs excreted viruses at 47 days after onset sequentially (Table 4, Fig 1).

**Table 4:** State of virus isolation for different groups of HFMD patients during different times of follow-up.

Time	EV-A71			CV-A16			Other EVs			Mixed EV-A71/CV-A16		
(days)	No. of follow-up patients	No. of patients with virus shedding	Positive rate	No. of follow-up patients	No. of patients with virus shedding	Positive rate	No. of follow-up patients	No. of patients with virus shedding	Positive rate	No. of follow-up patients	No. of patients with virus shedding	Positive rate
			(%)			(%)			(%)			(%)
0~	43	43	100	27	27	100	11	11	100	2	2	100
3~	43	38	88.4	27	27	100	11	11	100	2	1	50
5~	43	34	79.1	27	23	85.2	11	9	81.8	2	1	50
12~	43	24	55.8	27	21	77.8	11	7	63.6	2	1	50
19~	43	22	51.2	27	17	63	11	6	54.5	2	1	50
26~	43	15	34.9	27	17	63	11	6	54.5	2	1	50
33~	43	13	30.2	27	17	63	11	5	45.5	2	1	50
40~47	43	11	25.6	27	14	51.9	11	3	27.3	2	1	50

67 negative-culture samples (67/150) of HFMD probable cases were tested by RT-PCR. 9 stool specimens (9/67) of HFMD probable were positive for EV RNA, and identified as EV-71 (1) and CV-A16 (8). 290 negative-culture samples, which were all of negative-culture samples followed the last positive sample from 61 confirmed cases, were detected by RT-PCR. EV RNA appeared in the first negative sample followed the last positive sample of 9 confirmed cases (9/61). 9 of EV RNA were identified as 3 EV-A71, 3 CV-A16, 2 other EVs and 1 EV-A71/ CV-A16 mixed.

## Discussion

The most common human viruses in carriers or in those in an infectious state are HEVs. In this study, the 13% prevalence of EV and its distribution trends among different age groups of healthy persons were similar to those found in previous studies [25]. A variety of HEV genotypes were identified from the stool specimens of healthy persons in this study. Although the results were different from those found in previous studies conducted in China [18], a common feature was that the HEV-B species accounted for a large proportion (75–87%) of the population in Yunnan. Among them, nine serotypes (55.6%, 73/131) were related to HFMD [26–29], and no EV-A71 and CV-A16 were detected in healthy persons. The other two serotypes (CV-A4 and E-25) were detected at a low frequency in both healthy persons and patients with HFMD. In contrast, a large number of EV-A71 (51.81%) and CV-A16 (32.53%) isolates, which were the major pathogens of HFMD in the study area, were found in patients with HFMD. In addition, CV-A10 and CV-A6, which were the secondary causes of HFMD, did not appear in healthy subjects [4, 26–31].

Regarding the prevalence of EV-A71 and CV-A16 in healthy individuals, a wide range of positive rates was reported in different areas. Surveillance of EV prevalence in healthy children was performed from 2009–2013 in the Yunnan Province of China, and the results indicate that the positive rates of EV-A71 and CV-A16 were 0, except in 2012 when the rate of EV71 was 0.3% [32]. In Guangdong Province, the positive rates were 0.39% and 1.47% for EV-A71 and 0.23% and 0.74% for CV-A16 from stool specimens that were collected in 2009 and 2010 [20]. Prevalence rates of 0 for EV-A71 and 0.24% for CV-A16 during a HFMD outbreak in Shandong Province were reported in 2010 [17]. Additionally, EV-A71 had an isolation rate of 1.8% from stool samples that were collected from children in Shenzhen, China [18]. Studies conducted with children in Finland [33] and Norway [19, 25, 34] report EV-A71 positive rates of 0.3% and 1.4%, respectively, from stool samples. An overwhelming majority of studies demonstrate no or extraordinarily low positive rates of EV-A71 and CV-A16 among healthy children. However, Zhang *et al*. report the highest EV-A71 detection rate (15.0%) among healthy children [16], though the specimens were collected from kindergarteners during a HFMD epidemic. Previous studies and our study demonstrate that the predominant pathogens of HFMD, such as EV-A71, CV-A16, CV-A10, and CV-A6, are not commonly or rarely carried by healthy persons, and they may serve as a reservoir that maintains the circulation of HFMD viruses within a population or to limit transmission and make it sporadic.

In recent years, PCR has been widely used to detect EVs because it saves time and labor and is more sensitive than viral isolation in cell culture. Additionally, the EV isolation rate of 55.3% in stool specimens from probable HFMD cases in this study was less than the positive rate of 66% of EV nucleotides found in previous data [35]. Furthermore, RT-PCR can increase the rate of EV detection by up to 10% compared to cell culture [36], and positive of EV isolation in specimens indicates the presence of 100% live viruses with high titer. Therefore, a combination of findings using these two techniques may be more helpful in evaluating EV prevalence and shedding.

In mainland China, EV-A71 and CV-A16 are the main causative agents of HFMD, but in recent years, multiple enterovirus serotypes (*e*.*g*., CV-A10, CV-A6, and HEV-B) have been identified in patients with HFMD. Additionally, the number of HFMD cases caused by non-EV-A71 and non-CV-A16 viruses has increased in mainland China [37].

The phenomenon of intermittent excretion of EVs was also discovered in this study. This is similar to rotavirus [38] and polio virus. Similar to previous studies, we found that patients excreted EV-A71 and CV-A16, as well as other EVs, in their stools for a long period after onset. Various previous studies report that the durations of EV-A71 shedding in fecal samples is 6, 8, 10, or 11 weeks, respectively. The longest duration was 11 weeks. The 6-week duration applied to cases of CV-A16 after onset [15, 39–42]. Previous data shows that the positive rate of EV-A71 in stool specimens of a group with mild cases dropped to 50% at the end of the third week. For the group with severe cases, this occurred at the end of the fourth or fifth week, and for CV-A16, this occurred at the end of the second to fourth week after onset [15, 35, 40, 41]. A study also shows that the mild case group turned EV71-negative with a median shedding duration of 18 days [15]. This result is similar to ours, which was also 20 days.

Although the role of prolonged excretion in perpetuating epidemics of HFMD is uncertain, humans are the only known hosts for EVs, and patients who excrete HFMD pathogens for a long period after their recovery from the disease may be asymptomatic and have persistent infection, which contributes to the large epidemics that occur annually. In addition, the few patients who are asymptomatic carriers of pathogens of HFMD may serve as a reservoir that maintains the continuous circulation of EVs associated with HFMD among humans, as well as the environment, for future epidemics [15]. HFMD is predominantly transmitted via the fecal-oral route but can also spread through contact with virus-contaminated oral secretions, vesicular fluid, surfaces or fomites, and respiratory droplets [3]. Our previous study conducted in this area of the epidemic and other study demonstrated that contact with HFMD patients is a high risk factor for infection [43].

Theoretically, to thoroughly eliminate potential infection sources in communities, villages, childcare facilities, and schools, patients should be isolated from other susceptible children until they no longer excrete the virus, and the feces of these patients should be decontaminated. Unfortunately, these practices are difficult to achieve in practice. The isolation period of patients with HFMD that is recommended in national guidelines is approximately 2 week shorter than the duration of EVs that are associated with HFMD shedding in the stools of most patients. In recent years, the annual incidence of HFMD has still been surprisingly high in China despite public health efforts taken to prevent and control the disease including the early detection of patients to facilitate prompt isolation. The status of HFMD in China indicates that the effectiveness of public health measures currently used during HFMD epidemics is uncertain. At the moment, vaccines such as those for polio, EV-A71, and CV-A16 are successful at providing the most effective and economical means for disease control [44].

In summary, carriers of major causative agents of HFMD in healthy populations are uncommon. The excretion of EV-A71, CV-A16, and other EVs may last longer than 1 month after patients recover from HFMD, and a number of those recovering from HFMD may be asymptomatic pathogenic carriers of HFMD, serving as ongoing infection sources and reservoirs for HFMD in highly endemic areas of southwest China.
